# 44 Transcriptomic Analysis Reveals Compensatory Protective Role of Prokineticin-2 Against Pulmonary Inflammation After Severe Burn Injury

**DOI:** 10.1093/jbcr/irae036.044

**Published:** 2024-04-17

**Authors:** Yueqing Zhang, Xiaofu Wang, Juquan Song, Ravi S Radhakrishnan, Jun Yang

**Affiliations:** University of Texas Medical Branch at Galveston, Galveston, Texas; University of Texas Medical Branch at Galveston, Galveston, Texas; University of Texas Medical Branch at Galveston, Galveston, Texas; University of Texas Medical Branch at Galveston, Galveston, Texas; University of Texas Medical Branch at Galveston, Galveston, Texas

## Abstract

**Introduction:**

Severe burn injuries frequently result in multi-organ dysfunction, with the lungs being particularly susceptible, leading to substantial morbidity and mortality. However, the mechanisms underlying post-burn pulmonary injury remain to be elucidated. Our objective was to provide a comprehensive transcriptomic profile of lungs following severe burn injury to explore novel biomarker genes and potential therapeutic targets for burn injury induced lung diseases.

**Methods:**

A severe burn animal model was established in C57BL/6J mice by undergoing 30% total body surface area scald injury to produce a full-thickness burn. A total of twelve C57BL6 male mice (10-weeks-old) were divided into three groups (Sham, 3 days and 7 days post burn). Mouse lung tissues were harvested post-burn and the differential gene expression profiling was performed by RNA-Seq analysis. Gene ontology (GO) and Kyoto Encyclopedia of Genes and Genomes (KEGG) enrichment analyses were conducted to predicate the related pathological pathways and candidate genes. Quantitative RT-qPCR was employed to verify the gene expression and functions in both in vivo and in vitro settings (using human primary small airway epithelial cells, hSAECs). Statistical analysis was conducted using GraphPad Prism version 10.0.

**Results:**

Transcriptomic data revealed a total of 419 differentially expressed genes (DEGs) in both burned groups compared to the sham group (fold-change > 2 and p value < 0.05). Of these, 265 genes were uniquely changed at 3 days post burn, 87 genes were common, and 67 genes were regulated at 7 days post burn. Additionally, 306 DEGs were identified when comparing the 3-day and 7-day post-burn periods. Pathway analysis highlighted the top three enriched pathways in response to burn injury: Inflammation mediated by chemokine and cytokine signaling, Interleukin signaling and Apoptosis signaling. Notably, Prokineticin-2 (PK2) emerged as the most significantly altered gene in the lungs of burned mice. It was confirmed by qRT-PCR analysis that PK2 was initially detected at 3 days post burn and dramatically increased at 7 days post burn. Treatment of cultured hSAECs with recombinant human PK2 peptide resulted in decreased TNFa-induced inflammation responses (IL-6, IL-8, MMP9, and Grob) and reduced respiratory rhinoviral infection, suggesting the compensatory protective role of PK2 against burn-induced lung inflammation and infection.

**Conclusions:**

Our study unveils the distinctive gene expression profile in the lungs of scalded mice and identifies a paradigm for compensatory protective PK2 signaling. This finding may serve as a crucial foundation for future in-depth investigations into burn-induced lung injury.

**Applicability of Research to Practice:**

This research illuminates the protective role of PK2 against pulmonary inflammation following severe burn injuries, offering potential avenues for therapeutic interventions and improved patient care.

